# Zonulin and blood–brain barrier permeability are dissociated in humans

**DOI:** 10.1002/ctm2.965

**Published:** 2022-07-08

**Authors:** Charlotte M. Stuart, Aravinthan Varatharaj, Martin E. Winberg, Pascale Galea, Henrik B. W. Larsson, Stig P. Cramer, Alessio Fasano, Zaynah Maherally, Geoffrey J. Pilkington, Åsa V. Keita, Ian Galea

**Affiliations:** ^1^ Clinical Neurosciences, Clinical and Experimental Sciences, Faculty of Medicine University of Southampton Southampton UK; ^2^ Wessex Neurological Centre University Hospital Southampton NHS Foundation Trust Southampton UK; ^3^ Department of Biomedical and Clinical Sciences Linköping University Linköping Sweden; ^4^ Biomarker Discovery Unit Bio‐Rad Montpellier France; ^5^ Functional Imaging Unit, Department of Clinical Physiology and Nuclear Medicine Rigshospitalet Copenhagen Denmark; ^6^ Centre for Celiac Research and Treatment Massachusetts General Hospital Boston Massachusetts USA; ^7^ Cellular and Molecular Neuro‐Oncology Group University of Portsmouth Portsmouth UK

**Keywords:** blood–brain barrier, haptoglobin, permeability, zonulin

Dear Editor,

Zonulin, or prehaptoglobin‐2, mediates intestinal permeability in coeliac disease through the regulation of epithelial tight junctions.^1^ Tight junction breakdown at the blood–brain barrier (BBB) is a common pathological finding in neurological disease,^2^ and several in vitro and preclinical in vivo studies have suggested that zonulin plays a role in modulation of BBB permeability,^3^, ^4^, ^5^, ^6^ yet using multiple methods, we here consistently find that zonulin plays a negligible role in human BBB permeability.

Zonulin is a member of the MASP (mannose‐binding lectin‐associated serine protease) family of proteins, and elevated serum zonulin levels have been reported in a number of neurological conditions such as multiple sclerosis^7^ and Alzheimer's disease.^8^ The significance of zonulin upregulation in these neurological diseases is not certain. Specifically, it is not clear whether zonulin is an epiphenomenon, or has an effect on the brain, whether directly or mediated through the gut–brain axis.

To study the association between zonulin and BBB permeability in healthy individuals and patients with neurological disease, we employed two techniques to measure permeability across a range of molecular weights: *Q*_Alb_, or the quotient of cerebrospinal fluid to serum albumin (60 000 Da) in Study A and dynamic contrast‐enhanced magnetic resonance imaging with gadobutrol tracer (600 Da) in Study B (Supplemental Methods). Participant characteristics are shown in Table 1.

**TABLE 1:** Participant characteristics

	**Study A**		**Study B**	
	**Controls (*n* = 40)**	**Neurological disease** ^a^ **(*n* = 154)**	**Controls (*n* = 12)**	**Relapsing–remitting multiple scleroses (*n* = 11)**
**Age (years)**	50.5	53.4	31.3	43.4
**Sex (% female)**	57	47	67	73
**Haptoglobin phenotype (count)**				
*HP1‐1*	7 (17.5%)	25 (16.2%)	1 (8%)	1 (9%)
*HP2‐1*	18 (45.0%)	63 (40.9%)	7 (58%)	5 (45.5%)
*HP2‐2*	15 (37.5%)	66 (42.9%)	4 (33%)	5 (45.5%)
**Zonulin (ng/ml)**	63.0 (290.5)	58.5 (216.9)	.0 (314.4)	67.5 (323.8)
***Q*_Alb_**	.005 (.003)	.007 (.01)	–	–
***K_i_***	–	–	−.006 (.03)	.06 (.05)

*Note*: Age is given as the mean, zonulin, *Q*_Alb_ and *K_i_* are given as medians (interquartile range).

^a^ Diagnoses for participants with neurological disease in Study A included: inflammatory disease (*n* = 79), degenerative disease (*n* = 13), ischaemic disease (*n* = 13), normal pressure hydrocephalus (*n* = 9), infectious (*n* = 5), headache syndrome (*n* = 5), tumour (*n* = 2), structural (*n* = 2), epilepsy (*n* = 1), idiopathic (*n* = 1), hereditary neuropathy (*n* = 1), metabolic (*n* = 1), vascular (*n* = 1) and unknown (*n* = 21).

Out of a total of 217 cases (including people with neurological conditions and control individuals) across both studies, 58 (27%) individuals tested negative for serum zonulin using a novel enzyme‐linked immunosorbent assay (ELISA) (Supplemental Methods). Serum zonulin concentration followed a non‐Gaussian distribution with a range of 0–11 µg/ml. There were no effects of sex, age or disease status (disease vs. control) on zonulin concentrations (analysis of covariance, *F*(3,216) = 1.06, *p* = .37). In order to assess zonulin using a complementary dual approach, we determined the haptoglobin phenotype (Supplemental Methods) across all participants from both Studies A and B. As zonulin is a precursor of haptoglobin‐2, one would expect an increase in serum zonulin concentration with *HP2* allele dosage (*HP1‐1* < *HP2‐1* < *HP2‐2*), and this pattern was indeed observed (Figure S1), with a significant difference in zonulin among haptoglobin phenotypes (analysis of variance, *F*(2,216) = 43.8, *p* < .0001). However, it is important to note that 9 out of 34 *HP1‐1* cases tested positive for zonulin (Figure S1), indicating that the ELISA was cross‐reacting against other ZFP (zonulin family of proteins) members, as established previously with other zonulin ELISAs.^9^ This highlights the importance of adopting a dual approach, when assessing zonulin, by using both ELISA and haptoglobin phenotyping.

In Study A, *Q*_Alb_ was significantly higher with age (*p* = .0002), higher in males versus females (*p* < .0001) and higher in people with neurological disease versus healthy controls in univariable analysis (*p* = .0003, Figure 1A–C). A multivariable linear regression, controlling for age, sex and disease status, showed that serum zonulin did not associate with *Q*_Alb_ (*p* = .92, Table 2 and Figure 1D). Regressing *Q*_Alb_ on the presence or absence of the *HP2* allele (*HP2‐2* and *HP2‐1* individuals vs. *HP1‐1* individuals) instead of zonulin concentration, using the same covariates, also showed no association (*p* = .313, data not shown).

**FIGURE 1:**
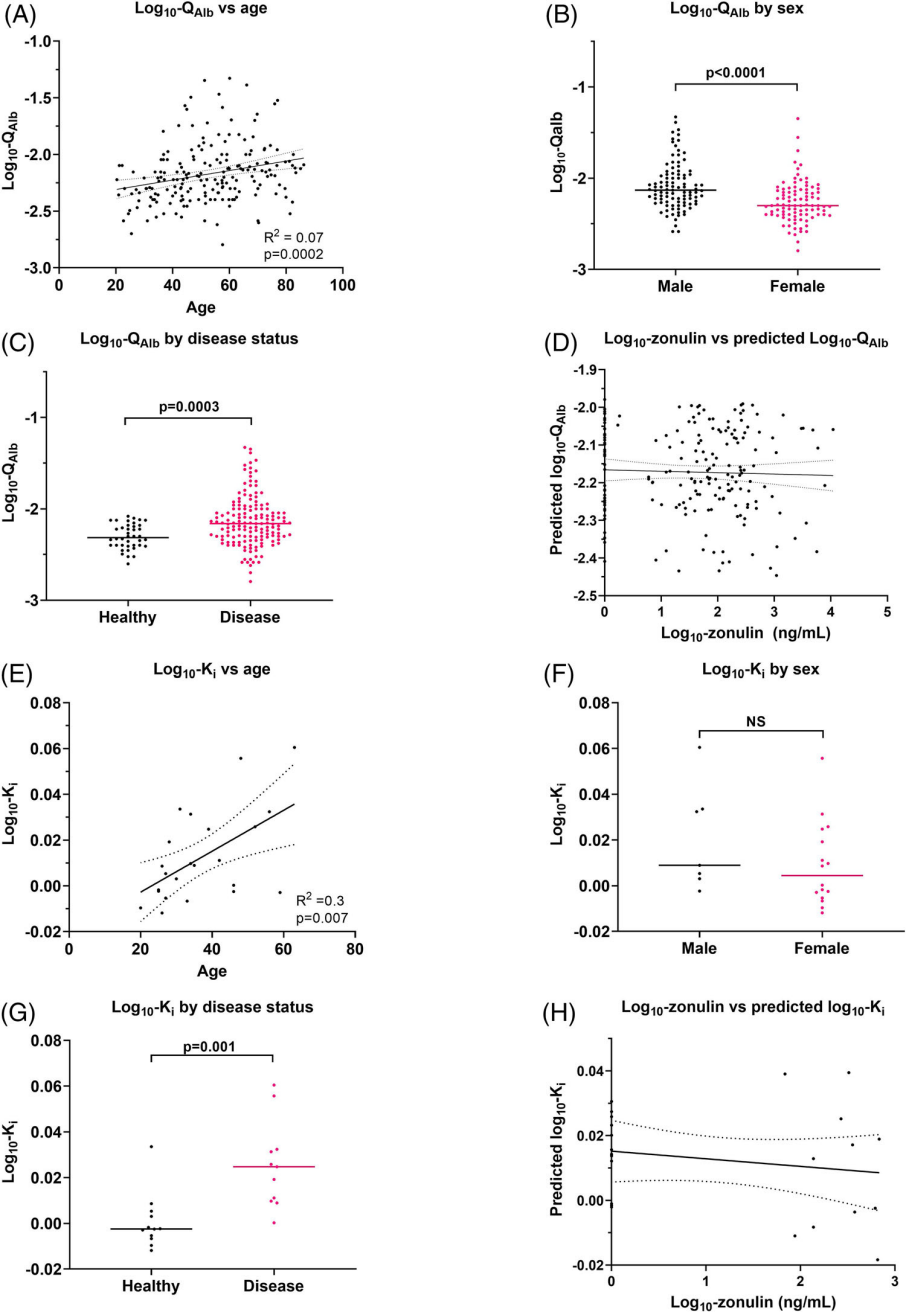
Study A employed *Q*_Alb_ as a human blood–brain barrier (BBB) permeability marker. *Q*_Alb_ was higher with age (A) and in males (B) and individuals with neurological disease (C), in univariable analyses. (D) Multivariable linear regression showed no relationship between zonulin and *Q*_Alb_. Study B used dynamic contrast‐enhanced magnetic resonance imaging (DCE‐MRI) to derive *K_i_* as a measure of human BBB permeability. *K_i_* was significantly higher with age (E), was not different between males and females (F) and was significantly higher in individuals with multiple sclerosis versus healthy individuals (G) in univariable analyses. (H) Multivariable linear regression showed no relationship between zonulin and *K_i_*. As *K_i_* in healthy individuals is close to zero, negative values may arise due to random noise. No positivity constraint was applied to the data. In (A), (D), (E) and (H), dashed lines represent 95% confidence intervals.

**TABLE 2:** Multivariable linear regression results

**Study A: *Q*_Alb_ as a marker of BBB permeability**							
	**Unstandardized coefficients**		**Standardized coefficients**			**95% confidence interval for *B***	
**Independent variable**	***B***	**Std. error**	**Beta**	***t***	**Sig**.	**Lower bound**	**Upper bound**
**(Constant)**	−2.354	.076		−30.976	.000	−2.504	−2.204
**Log_10_‐zonulin (ng/ml)**	−.002	.016	−.007	−.106	.916	−.033	.03
**Sex**	−.144	.035	−.279	−4.086	**.000**	−.214	−.075
**Age**	.003	.001	.168	2.475	**.014**	.001	.005
**Disease status** ^a^	.142	.042	.221	3.379	**.001**	.059	.224

**Study B: using *K_i_* as a marker of BBB permeability**							
	**Unstandardized coefficients**		**Standardized coefficients**			**95% confidence interval for *B***	
**Independent variable**	***B***	**Std. Error**	**Beta**	***t***	**Sig**.	**Lower bound**	**Upper bound**
**(Constant)**	−.002	.011		−.185	.855	−.024	.02
**Log_10_‐zonulin (ng/ml)**	−.005	.002	−.305	−2.106	.05	−.01	.00
**Sex**	−.013	.006	−.312	−2.175	**.043**	−.026	.00
**Age**	.001	.00	.321	1.92	.071	.00	.001
**Disease status** ^b^	.021	.006	.529	3.202	**.005**	.007	.034

*Note*: For Study A, using *Q*_Alb_ as a marker of BBB permeability: model fit: *F*(4,193) = 11.6, *p* < .0001, *R* ^2^ = .20, adjusted *R* ^2^ = .18. For Study B, using *K_i_* as a marker of BBB permeability: model fit: *F*(4,22) = 7.99, *p* = .001, *R* ^2^ = .64, adjusted *R* ^2^ = .56. Bold values indicate *p* < .05.

Abbreviation: BBB, blood–brain barrier.

^a^ Healthy versus neurological disease.

^b^ Healthy versus multiple sclerosis.

In Study B, *K_i_* was significantly higher with age (*p* = .007), was not different between males versus females (*p* = .23) and was significantly higher in people with multiple sclerosis versus healthy individuals in univariable analysis (*p* = .001, Figure 1E–G). A multivariable linear regression, controlling for sex, disease status and age, showed that serum zonulin was not positively associated with *K_i_* (Table 2 and Figure 1H). Regressing *K_i_* on the presence or absence of the *HP2* allele (*HP2‐2* and *HP2‐1* individuals vs. *HP1‐1* individuals) instead of zonulin concentration also showed no association (*p* = .50, data not shown). Most circulating mediators relevant to pathology, such as cytokines, lipopolysaccharide, viral nucleic acids, complement components, kinins, prostaglandins, hormones and neuroactive monoamines,^2^ have molecular weights below 60 kDa. It is currently not technically possible to measure the permeability of the human BBB to larger molecular weight substances in vivo,^2^ yet this is important for immunoglobulin G which has a molecular weight of 150 kDa. Hence, we examined BBB permeability to larger molecules using fluoresecent dextrans (70 and 150 kDa) in a well‐established human brain endothelial cell line (hCMEC/D3) BBB model (Supplemental Methods).

The effect of zonulin on the permeability of the hCMEC/D3 monolayer to 70 and 150 kDa fluorescent dextrans was assessed at 1‐h intervals up to 6 h after treatment with recombinant zonulin. A 1:1 mixture of the cytokines TNF‐α (tumour necrosis factor‐alpha) and IFN‐γ (interferon‐gamma) was used as a positive control. Compared to vehicle control wells, TNF‐α and IFN‐γ significantly increased the permeability of the hCMEC/D3 monolayer to the 70‐kDa dextrans (Figure 2A,C) and 150‐kDa dextrans (Figure 2B,D). Monolayers treated with zonulin showed no difference in permeability to the 70‐kDa dextrans (Figure 2A,C) and 150‐kDa dextrans (Figure 2B,D) compared with controls.

**FIGURE 2:**
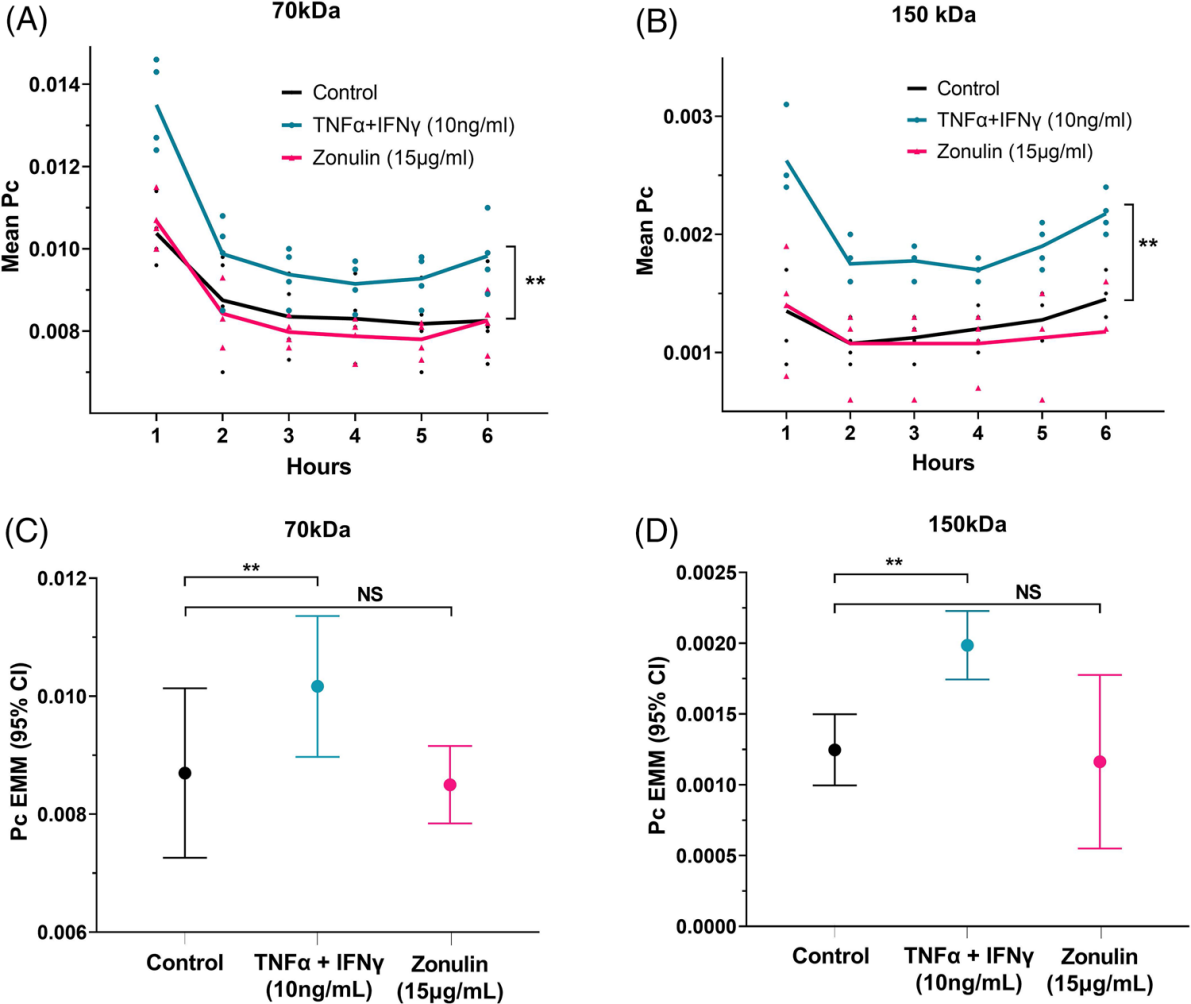
Permeability of human cerebral microvascular endothelial cell (hCMEC/D3) monolayers to 70‐kDa (A) and 150‐kDa (B) dextrans in the presence of vehicle (black), tumour necrosis factor‐alpha (TNF‐α) + interferon‐gamma (IFN‐γ) as positive control (blue) or zonulin (pink) at hourly intervals over a 6‐h period. Two‐way repeated measures analysis of variance (ANOVA) revealed that there was a significant main effect of TNF‐α + IFN‐γ on the *P*_c_ for the 70‐kDa dextran (*F*(1,3) = 268.4, *p* < .001, *η*_p_^2^ = .989) and 150‐kDa dextran (*F*(1,3) = 196.6, *p* < .001, *η*_p_^2^ = .985), but there was no effect of zonulin on the *P*_c_ for either 70‐kDa dextran (*F*(1,3) = .34, *p* = .601, *η*_p_^2^ = .102) or 150‐kDa dextran (*F*(1,3) = .152, *p* = .723, *η*_p_^2^ = .048). (C and D) The estimated marginal mean (EMM) of the *P*_c_ (controlling for time in the two‐way repeated measures ANOVA) was higher after cytokine treatment, but similar between zonulin and vehicle‐treated wells. NS, not significant. **p < .001. All experiments were repeated four times (*n* = 4), each with triplicate wells per condition.

This is the first study to examine the role of zonulin in BBB permeability in humans. A major strength of this work, important in confirming the absence of a significant contribution of zonulin to BBB regulation, is the robustness of findings using different methodologies. Still, it remains possible that local and/or transient changes in concentrations of zonulin at brain capillary surfaces are not well represented by either circulating zonulin levels or haptoglobin phenotype. The simplistic in vitro model of the BBB used does not fully recapitulate the anatomy and physiology of the living BBB, and future studies should aim to replicate results using three‐dimensional all‐human multicellular BBB models and more sophisticated methods for assessing BBB permeability.

Although preclinical studies suggested that zonulin has potential to regulate BBB permeability,^3^, ^4^, ^5^, ^6^ we find no evidence for a significant contribution of zonulin in humans, using a number of technical approaches to account for zonulin and to quantify BBB permeability. This is an important negative finding and suggests that the association of serum zonulin levels with clinical manifestations in various neurological diseases^7^, ^8^ is unlikely to be mediated by a direct effect of zonulin on BBB permeability. Other indirect mechanistic pathways such as gastrointestinal permeability linked with the gut–brain axis are more likely to be responsible, as exemplified by zonulin transgenic mice which display neurological abnormalities improved by antibiotic depletion of gut microbiota.^10^ Future studies should further investigate these pathways and the relationship between zonulin and the severity of neurological disease.

## CONFLICT OF INTEREST

None of the authors have any potential competing interests.

## Supporting information


**Figure S1** Zonulin serum concentration (mean ± SD) was significantly different among haptoglobin phenotypes (Studies A and B, ANOVA, *F*(2,232) = 51.13, *p* < .0001).
**Figure S2** The HP2 gene arose from a duplication of complement control protein (CCP) domain region of the HP1 gene. The region unique to the prehaptoglobin‐2 (zonulin) sequence is shown.
