# Supplementary material for: Zonulin and blood–brain barrier permeability are dissociated in humans
Source: Clin Transl Med. 2022 Jul 8;12(7):e965. doi: 10.1002/ctm2.965 (PMC9270641; doi:10.1002/ctm2.965)
Supplement: Supplementary file 1 — Figure S1 Zonulin serum concentration (mean ± SD) was significantly different among haptoglobin phenotypes (Studies A and B, ANOVA, F(2,232) = 51.13, p < .0001). Figure S2 The HP2 gene arose from a duplication of complement control protein (CCP) domain region of the HP1 gene. The region unique to the prehaptoglobin‐2 (zonulin) sequence is shown. [file CTM2-12-e965-s001.docx]

**Supplemental material for:**

**Zonulin and blood-brain barrier permeability are dissociated in humans**

Charlotte M. Stuart^1^, Aravinthan Varatharaj^1,2^, Martin E. Winberg^3^, Pascale Galea^4^, Henrik B.W. Larsson^5^, Stig Cramer^5^, Alessio Fasano^6^*,* Zaynah Maherally^7^, Geoffrey J. Pilkington^7^, Åsa V Keita^3^*,* Ian Galea^1,2^

^1^ Clinical Neurosciences, Clinical and Experimental Sciences, Faculty of Medicine, University of Southampton, UK

^2^ Wessex Neurological Centre, University Hospital Southampton NHS Foundation Trust, UK

^3^ Department of Biomedical and Clinical Sciences, Linköping University, 58185 Linköping, Sweden

^4^ Biomarker Discovery Unit, Bio-Rad, Montpellier, France

^5^ Functional Imaging Unit, Department of Clinical Physiology and Nuclear Medicine, Rigshospitalet, Copenhagen, Denmark.

^6^ Centre for Celiac Research and Treatment, Massachusetts General Hospital, Boston, USA

^7^ Cellular and Molecular Neuro-Oncology Group, University of Portsmouth, UK

# **SUPPLEMENTAL METHODS**

## **1 Clinical studies**

Participants were recruited into two separate studies which assessed BBB permeability in two ways. Study A utilized the quotient of cerebrospinal fluid (CSF) to serum albumin (Q_Alb_)^1^ since albumin is not synthesized within the CNS, while Study B used dynamic contrast-enhanced magnetic resonance imaging (DCE-MRI) to derive the influx constant K_i_, an index of BBB permeability.^2^ Both studies were covered by National Research Ethics Service approval (11/SC/0204 and 12/SC/0176 respectively) and institutional research ethics approval (ERGO 41084.A1 and 5562 respectively).

1.1 Study A: Q_Alb_

In Study A, the CSF/serum Q_Alb_ was determined in participants with unselected neurological diseases (n=154) presenting sequentially to the regional neurological service and control individuals (n=40) with normal CSF composition. Demographics and clinical characteristics are provided in Table 1. Serum albumin was measured by detection of a coloured albumin-Bromocresol Purple complex, which absorbs at 600 nm and is directly proportional to the albumin concentration in the sample. Albumin was measured in the CSF using rate nephelometry.

1.2 Study B: DCE-MRI

In Study B, DCE-MRI was used to assess BBB permeability in normal appearing white matter in participants with relapsing-remitting multiple sclerosis (n=11), diagnosed according to the 2017 MacDonald criteria,^3^ and healthy control individuals with no neurological disease (n=12), as previously described.^2^ Demographics and clinical characteristics are provided in Table 1. Briefly, DCE-MRI was conducted on a 3 Tesla magnetic resonance scanner (Skyra, Siemens, Erlangen, Germany) using a 20-element phased-array head coil and a 3D spoiled gradient echo sequence (FLASH) covering the whole brain with a spatial resolution of 1.3 × 1.3 × 5.0mm^3^, acquiring 300 dynamic frames with a time resolution of 3.2 seconds. Intravenous contrast injection Gadovist (gadobutrol) at a dose of 0.05mmol/kg was given after the tenth acquisition. The mean unidirectional influx constant K_i_ was computed using the Patlak method^4^ in normal-appearing white matter regions of interest after brain extraction using BET^5^ and tissue segmentation using FAST.^6^ Partial volume effects were minimized by applying a threshold of 100% white matter probability to the tissue segmentation probability map.

## **1.3 Zonulin assay**

Zonulin was assessed in two ways. First, we measured serum zonulin levels at the time of BBB permeability assessment using a non-commercial enzyme-linked immunosorbent assay (ELISA), developed and validated to measure zonulin.^7,8^ Secondly, we determined the individual’s haptoglobin phenotype; since zonulin is the unprocessed precursor form of haptoglobin-2, we reasoned that people homozygous for haptoglobin-1^9^ would not produce zonulin.

**1.3.1 Zonulin ELISA**

Zonulin is only one member of the ZFP family of proteins, which share structural similarities as evidenced by cross-reactivity in several ELISAs. For example, a commercially available zonulin ELISA from Immunodiagnostik has been found to measure properdin which like pro-haptoglobin-2, is a member of the MASP (mannose-binding lectin-associated serine protease) family.^10^ Another commercially available zonulin ELISA from Cusabio was also recently found to be non-specific for pro-haptoglobin-2.^8^ The ELISA used in the present study was developed by BioRad^7^ and is not yet commercially available, yet it is clearly superior to the ones from Immunodiagnostik and Cusabio in terms of concordance with haptoglobin phenotype (compare Supplemental Figure 1 in this work with Figure 1 in Scheffler et al^10^ and with Table 1 in Meira de-Faria F et al^8^). The antibodies used in the BioRad ELISA were developed by immunizing mice with recombinant zonulin as well as peptides unique to prehaptoglobin-2 within the alpha duplication region, and were selected from thousands of clones after a combinatorial process of iterative sandwich ELISAs against precursor and mature forms of haptoglobin-1 and 2 (Supplemental Figure 2), surface plasmon resonance and epitope mapping.^7^ A 96-well plate (Nunc-Immuno microwell, MaxiSorp) was coated with 2µg/mL primary mouse monoclonal anti-human prehaptoglobin-2 (zonulin) capture antibody (50µL/well, 13D11-G7-B10, BioRad) and incubated overnight at 4˚C. Antibody was removed and the plate blocked with blocking buffer (phosphate-buffered saline (PBS) + 3% bovine serum albumin (BSA)) for 1h at room temperature. Serum samples (1:5 dilution) and standards were prepared in sample diluent (PBS + 0.1% Tween-20 + 0.1% BSA + 0.3mg/mL mouse IgG (I5381, Sigma, reconstituted in sterile 0.9% saline)). Blocking buffer was removed, and samples and standards were added (50µL/well) and incubated at room temperature for 2h. The plate was then washed three times (PBS + 0.1% tween-20), 2µg/mL of detection antibody added (50µL/well, biotinylated monoclonal anti-zonulin conjugate antibody,11G3-G9-G8, Biorad) diluted in conjugate diluent (PBS + 0.1% Tween-20 + 0.1% BSA) and incubated at room temperature for 1h. Following three washes to remove any unbound secondary antibody, horseradish peroxidase-conjugated streptavidin (S2438, Sigma) was added (1:10 000 dilution, 100µL/well). After three more washes, a TMB substrate solution (BioRad) was added (100µL/well) and colour left to develop in the dark for 15mins. Colour development was stopped using 10% v/v sulphuric acid (50µL/well) and the colour intensity measured using absorbance at 450nm. The concentration of zonulin in the samples was calculated using the standard calibration curve.

**1.3.2 Haptoglobin phenotyping**

Non-reducing western blot was employed to determine haptoglobin phenotype. Samples were diluted in sample buffer (without β-mercaptoethanol) and run on a gradient tris-glycine gel (Invitrogen Novex™ Wedgewell™ 4-12%) in Laemmli running buffer (25mMol Tris, 192mMol Glycine and 0.1% SDS) at 120V for 1.5h. After separation protein was transferred to a polyvinylidene difluoride (PVDF) membrane (TransBlot Turbo, Bio-Rad) and blocked in TBST (tris(hydroxymethyl)aminomethane-buffered saline (TBS) and 0.1% Tween, pH 8.0) with 3% Bovine Serum Albumin (BSA). The membrane was then incubated with primary antibody (1:5000 dilution, rabbit anti-haptoglobin, Sigma) for 1h at room temperature. The membrane was washed three times for 5mins in TBST and incubated for 45mins in secondary antibody (1:2000 dilution, goat anti-rabbit Alexa Fluor 488, Thermofisher). The membrane was washed again and imaged on a Bio-Rad ChemiDoc™ MP imager using blue epi illumination and auto-exposure.

## **2 *In vitro* BBB model**

2.1 Cell line

Human cerebral microvascular endothelial cells (hCMEC/D3),^11^ immortalized with hTERT catalytic subunits and Simian vacuolating virus 40 large T antigens were donated by Dr Pierre-Olivier Couraud (Institut Cochin, INSERM, Paris, France). This cell line has been specifically validated as a model of the human BBB. ^11^ Cells (passage 32-35) were grown without antibiotics in endothelial basal medium 2 (EBM-2, Lonza, Basel Switzerland) supplemented with SingleQuots and 2% foetal bovine serum (FBS, Lonza), at 5% CO_2_ atmosphere, 37°C incubator.

2.2 BBB model preparation

Transwell inserts (6.5mm, 0.4 µm pores, PET, Corning, fits 24-well plate) were coated with Collagen 1 (Merck) at 50µg/ml in 0.02M acetic acid for 1h at 37˚C prior to cell seeding. The transwells were then washed three times in sterile distilled water and hCMEC/D3 cells seeded onto the apical side of the transwells at a seeding density of 7.5 x 10^4^ cells/insert. Cells were grown for ~30hor until cells became confluent. Cell confluency was assessed by transendothelial electrical resistance (TEER) using the Endohm-6 and EVOM2 (World Precision Instruments). Once cells were confluent (TEER >10Ω/cm^2^), cell culture media was changed to EBM-2 without the growth factors (i.e. without hEGF, hFGF, VEGF and R3-IGF-1) and left for at least 24h. The cytokines TNF-α (tumour necrosis factor-alpha) and IFN-γ (interferon-gamma) (ThermoFisher Scientific) were used as positive controls to induce barrier leakiness. They were added at a final concentration of 10ng/mL for 24h before the permeability assay.

2.3 Permeability assay

All media used in the permeability assays was 2% FBS-supplemented EBM-2 without phenol-red (Lonza) to eliminate interference with fluorescence measurement. Recombinant zonulin synthesized in a baculovirus system as previously described,^12^ was added to the apical compartment at a final concentration of 15µg/mL along with 1mg/mL fluorescein isothiocyanate (FITC)-labelled 70kDa dextran (Sigma-Aldrich) and 1mg/mL Antonia Red-labelled 150kDa dextran (Merck). Dextrans (1mg/mL) were also added to the negative and positive control wells. The dose of zonulin was decided *a priori* to reflect the clinical situation of increased circulating zonulin levels. In the largest series of normal healthy individuals, the highest serum zonulin level was 3.2µg/mL.^7^ Since serum zonulin may increase by a factor of 4-5 in patients with increased gut permeability,^13^ we selected a working zonulin concentration of 15µg/mL. Within each experiment each well condition was performed in triplicate, and the experiment was repeated four times (n=4).

Permeability of the dextrans across the cell monolayer was assessed at 1h intervals for 6h by sampling the basolateral compartment in duplicate for fluorescence measurement at λ_exc_ 485nm and λ_em_ 525nm for FITC and λ_exc_ 583nm and λ_em_ 602nm for Antonia Red using a fluorescence reader (Fluorstar, BMG Labtech). Phenol red free media served as a blank.

The permeability coefficient (Pc) was calculated as previously described.^14,15^ Briefly the clearance or permeability-surface area product (PS) was calculated for the filter on its own (PS_F_), and for the combination of endothelium and filter (PS_E+F_), according to the formula: PS (in ml/h) = (C2 x VBL)/(C1)]/T, where C1 = initial fluorescence signal in the apical chamber; C2 = fluorescence signal in the basolateral chamber; VBL = volume of the basolateral compartment; T = time (h). The PS for the endothelial cell layer (PS_E_) is given by 1/PS_E_ = 1/PS_E+F_ – 1/PS_F_.^14^ The endothelial permeability coefficient (Pc, in cm^2^/hr) was derived by dividing the PS with the membrane surface area (in cm^2^).

## **3 Statistics**

Analyses were performed in SPSS Statistics version 25 (IBM) and figures prepared using Graphpad Prism version 8. Descriptive statistics are shown as median (interquartile range, IQR) or mean (standard deviation, SD). Data distribution was determined graphically and using the Kolmogorov-Smirnov test. Non-parametric data were log_10_-transformed (log_10_x=log_10_(x+1), to avoid error values when the variable value was zero). Group comparisons were performed using Student’s t-test or analysis of variance or covariance (ANOVA or ANCOVA respectively). Multivariable linear regression models were used to analyse the association between BBB permeability indices and zonulin. A p value of <0.05 was considered statistically significant.

## **4 Data availability**

The data that support the findings of this study are available from the corresponding author upon reasonable request, subject to institutional and national ethical approvals and regulations.

# **SUPPLEMENTAL FIGURES**

**Supplemental Figure 1.** Zonulin serum concentration (mean ± SD) was significantly different between haptoglobin phenotypes (Studies A & B, ANOVA, F(2,232)=51.13, p<0.0001).


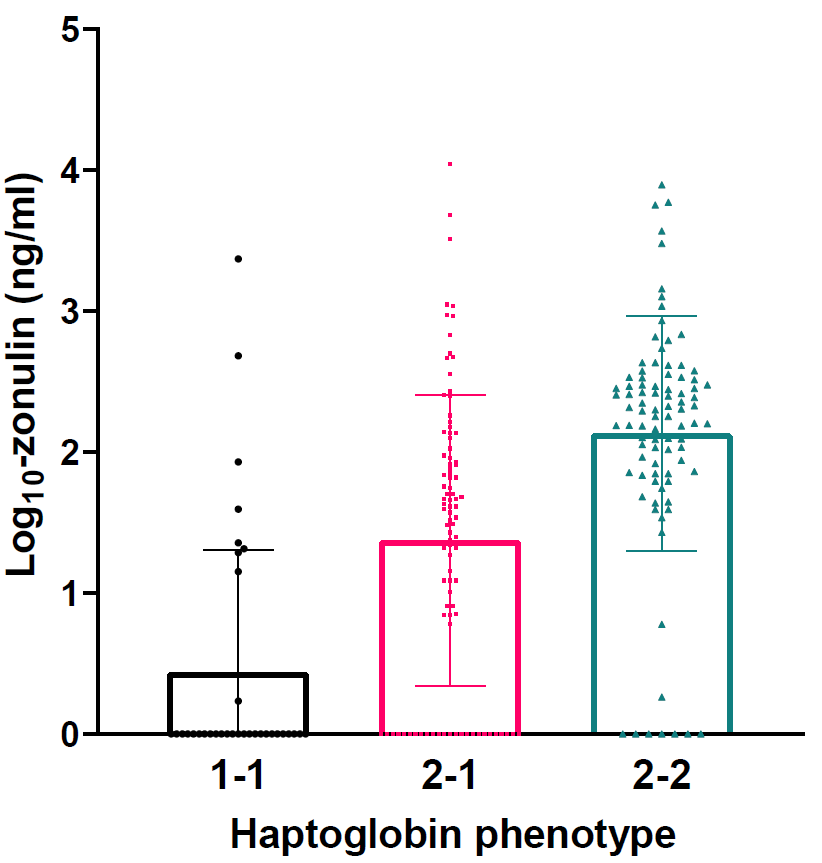


**Supplemental Figure 2.** The HP2 gene arose from a duplication of complement control protein (CCP) domain region of the HP1 gene. The region unique to the prehaptoglobin-2 (zonulin) sequence encompasses the CCP1/CCP2 domain and the alpha/beta chain cleavage site, distinguishing it from mature haptoglobin-2, prehaptoglobin-1 and mature haptoglobin-1


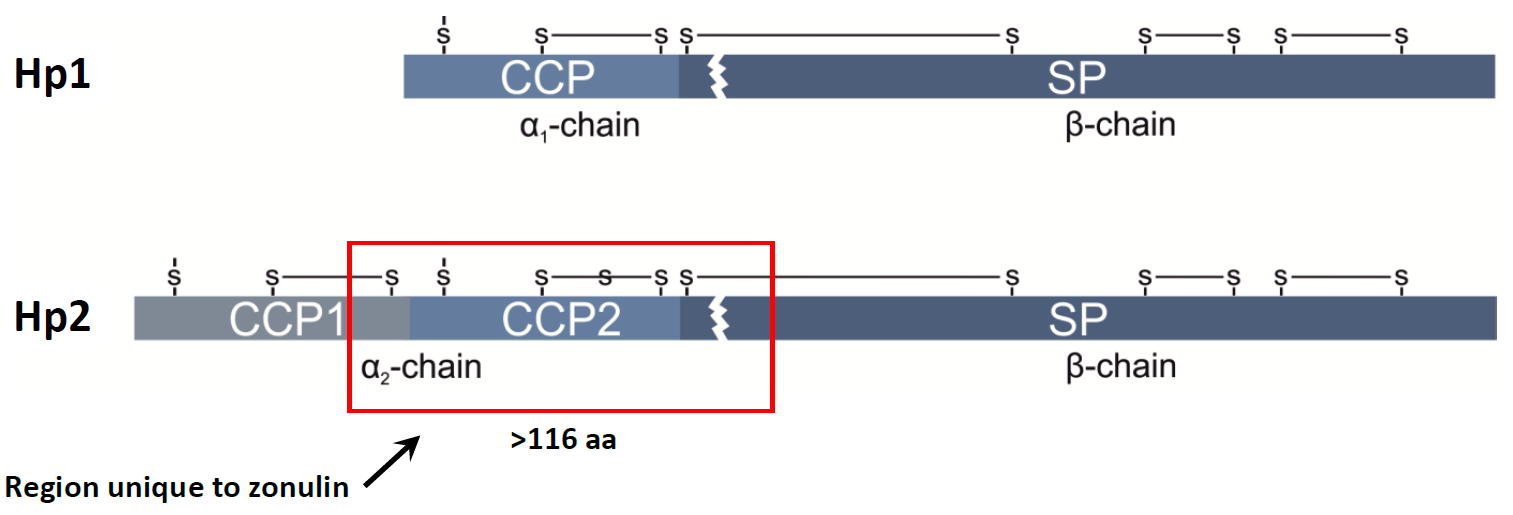


# **REFERENCES**

1. Thompson EJ. *Different blood–CSF barriers*. In: Proteins of the cerebrospinal fluid: analysis & interpretation in the diagnosis and treatment of neurological disease. 1^st^ ed. Academic Press Elsevier; 2005:43-63.

2. Varatharaj A, Liljeroth M, Darekar A, Larsson HBW, Galea I, Cramer SP. Blood-brain barrier permeability measured using dynamic contrast-enhanced magnetic resonance imaging: a validation study. *J Physiol*. 2019;597(3):699-709. doi:10.1113/jp276887

3. Thompson AJ, Banwell BL, Barkhof F, et al. Diagnosis of multiple sclerosis: 2017 revisions of the McDonald criteria.*Lancet Neurol*. 2018;17(2):162-173. doi:<https://doi.org/10.1016/S1474-4422(17)30470-2>

4. Patlak CS, Blasberg RG, Fenstermacher JD. Graphical evaluation of blood-to-brain transfer constants from multiple-time uptake data. *J Cereb Flow Metab*. 1983;3(1):1-7. doi:10.1038/jcbfm.1983.1

5. Smith SM. Fast robust automated brain extraction. *Human Brain Mapping*. 2002;17(3):143-155. doi:<https://doi.org/10.1002/hbm.10062>

6. Zhang Y, Brady M, Smith S. Segmentation of brain MR images through a hidden Markov random field model and the expectation-maximization algorithm. *IEEE Trans Med Imaging*. 2001;20(1):45-57. doi:10.1109/42.906424

7. Flanagan JJ, Arjomandi A, Delanoy ML, et al. Development of monoclonal antibodies to pre-haptoglobin 2 and their use in an enzyme-linked immunosorbent assay (ELISA).*J Immunol Methods*. 2014;406:34-42. doi:10.1016/j.jim.2014.02.009

8. Meira de-Faria F, Bednarska O, Ström M, Söderholm JD, Walter SA, Keita Å V. Colonic paracellular permeability and circulating zonulin-related proteins. *Scand J Gastroenterol*. 2021;56(4):424-431. doi:10.1080/00365521.2021.1879247

9. Langlois MR, Delanghe JR. Biological and clinical significance of haptoglobin polymorphism in humans. *Clin Chem*. 1996;42(10):1589-600.

10. Scheffler L, Crane A, Heyne H, et al. Widely Used Commercial ELISA Does Not Detect Precursor of Haptoglobin2, but Recognizes Properdin as a Potential Second Member of the Zonulin Family. *Front Endocrinol (Lausanne)*. 2018;9:22-22. doi:10.3389/fendo.2018.00022

11. Weksler BB, Subileau EA, Perriere N, et al. Blood-brain barrier-specific properties of a human adult brain endothelial cell line. *FASEB j*. 2005;19(13):1872-1874. doi:10.1096/fj.04-3458fje

12. Tripathi A, Lammers KM, Goldblum S, et al. Identification of human zonulin, a physiological modulator of tight junctions, as prehaptoglobin-2. Article. *Proc Natl Acad Sci U S A*. 2009;106(39):16799-16804. doi:10.1073/pnas.0906773106

13. Sapone A, de Magistris L, Pietzak M, et al. Zonulin upregulation is associated with increased gut permeability in subjects with type 1 Diabetes and their relatives. *Diabetes*. 2006;55(5):1443-1449. doi:10.2337/db05-1593

14. Dehouck M-P, Jolliet-Riant P, Brée F, Fruchart J-C, Cecchelli R, Tillement J-P. Drug Transfer Across the Blood-Brain Barrier: Correlation Between In Vitro and In Vivo Models.*J Neurochem*. 1992;58(5):1790-1797. doi:<https://doi.org/10.1111/j.1471-4159.1992.tb10055.x>

15. Tai LM, Holloway KA, Male DK, Loughlin AJ, Romero IA. Amyloid-β-induced occludin down-regulation and increased permeability in human brain endothelial cells is mediated by MAPK activation.*J Cell Mol Med* . 2010;14(5):1101-1112. doi:<https://doi.org/10.1111/j.1582-4934.2009.00717.x>
